# Invasive zebra mussel (*Dreissena polymorpha*) threatens an exceptionally large population of the depressed river mussel (*Pseudanodonta complanata*) in a postglacial lake

**DOI:** 10.1002/ece3.6243

**Published:** 2020-04-12

**Authors:** Małgorzata Ożgo, Maria Urbańska, Philipp Hoos, Hannes K. Imhof, Małgorzata Kirschenstein, Julia Mayr, Florian Michl, Rafał Tobiasz, Marie von Wesendonk, Stefan Zimmermann, Juergen Geist

**Affiliations:** ^1^ Department of Evolutionary Biology Kazimierz Wielki University Bydgoszcz Poland; ^2^ Department of Zoology Poznań University of Life Sciences Poznań Poland; ^3^ Aquatic Systems Biology Unit Technical University of Munich Freising Germany; ^4^ Aeronautics Faculty Polish Air Force University Dęblin Poland; ^5^ SharkDive Złocieniec Poland

**Keywords:** biodiversity conservation, biofouling, endangered species, freshwater habitat, invasion, unionid, zebra mussel

## Abstract

1. Freshwater mussels are in decline worldwide, with the depressed river mussel *Pseudanodonta complanata* being one of the rarest and most endangered species in Europe. Invasive mussels are suspected to be an important factor of decline, but there is little information on their interaction with native species.
2. This study analyzed densities, depth distribution, and individual sizes and weights in one of the largest known populations of *P. complanata* in Europe in relation to the co‐occurring invasive zebra mussel *Dreissena polymorpha* and other mussel species*,* using a systematic transect analysis.
3. *Pseudanodonta complanata* was the dominant unionid species in Lake Siecino reaching densities of up to 26 ind/m^2^, with half of the specimens found at a water depth of 2.0–4.0 m. Densities were highest on sandy substrates in areas of underwater currents. In contrast, 67% of native *Unio tumidus* were found at depths < 1 m, indicating different habitat preference.
4. In the study area, 91% of *P. complanata*, 92% of *U. tumidus*, and all *Anodonta* individuals were fouled by *D. polymorpha*. The dreissenid:unionid mass ratio (mean ± *SD*; maximum) was 0.43 ± 0.56; 4.22 and 0.86 ± 1.87; 8.76 in *P. complanata* and *U. tumidus*, respectively. *Pseudanodonta complanata* fouled with *D. polymorpha* were impaired in their anchoring capability and had shell deformations potentially affecting shell closing and filtration activity. Fouling intensity was negatively correlated with unionid density, potentially leading to accelerated population declines.
5. The observed adverse effects of invasive zebra mussels on the depressed river mussel and the difficulties in eradicating established populations of invasive mussels suggest that *D. polymorpha* should be considered a serious threat to *P. complanata*. Therefore, the further spread of zebra mussels into habitats with native unionids needs to be avoided by all means.

## Graphical abstract

This study revealed adverse effects of invasive zebra mussels on native mussel communities. An exceptionally large population of endangered *Pseudanodonta complanata* was discovered and found to experience fouling by zebra mussel. This resulted in shell deformation as well as impaired anchoring within the substrate.

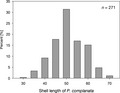

## 1 INTRODUCTION

Due to their important ecosystem functions (e.g., Lummer, Auerswald, & Geist, 2016; Vaughn, 2018) and their ongoing declines (Lopes‐Lima et al., 2017), conservation, and restoration of freshwater mussel populations are high on the agenda in Europe and elsewhere (e.g., Geist, 2011, 2015; Geist & Hawkins, 2016). To date, most of the scientific work on freshwater mussels and the threats to them have been focused on charismatic species such as the freshwater pearl mussel (*Margaritifera margaritifera*; see e.g., Geist, 2010; Boon et al., 2019), whereas there is less knowledge on the habitat requirements and threats of equally or even more rare species such as the compressed river mussel *Pseudanodonta complanata*.

*Pseudanodonta complanata* inhabits lotic environments, including lowland and mountainous rivers, large drains, and canals (Bonk, 2019; Killeen, Aldridge, & Oliver, 2004) and has also been reported from some lakes (Van Damme, 2011). Its geographical range extends across most of Europe, but its populations are usually small, scattered, and isolated. Most often, it co‐occurs with other unionids but is the least abundant species, usually contributing to less than 5% of specimens in unionid assemblages (Piechocki & Wawrzyniak‐Wydrowska, 2016; Zettler, 1998, 1999). Based on a small number of population assessments, strong population declines and fragmentation of *P. complanata* distribution were recorded (Skidmore, Leach, Hoffman, Amos, & Aldridge, 2010; Van Damme, 2011; Zając, 2009). Further declines are predicted under climate change scenarios (Gallardo & Aldridge, 2013). *Pseudanodonta complanata* is currently listed as vulnerable in the IUCN Red List, is legally protected in Germany and Poland, and is a species of conservation priority on the UK Biodiversity Action Plan.

In 2015, we discovered one of the largest known populations of this species in Lake Siecino in northern Poland (Ożgo, 2016), a postglacial lake with a size of 743 ha. This provided an excellent opportunity to study population characteristics and habitat use of this little known species. The invasive zebra mussel *Dreissena polymorpha* was introduced into this lake, and its abundant occurrence has been observed since the early 1980s (RT, pers. obs.); the initial time of invasion is unknown. *Dreissena polymorpha* attaches with byssal threads to all kinds of hard substrates, including mussel shells (Mackie, 1991). Generally, invasive mussel species have been proposed to infer with native mussels (e.g., Strayer, 1999), but there are only few examples where this interaction has been systematically studied. For instance, the Chinese pond mussel *Sinanodonta woodiana* was found to potentially outcompete European *Anodonta* species by a broader host fish use, faster metamorphosis and higher recruitment success as evident from both laboratory (Huber & Geist, 2019) and field studies (Urbańska, Kirschenstein, Obolewski, & Ożgo, 2019). Adverse effects of the globally invasive *D. polymorpha* on the physiological condition of native mussels (Sousa, Pilotto, & Aldridge, 2011) as well as on population declines (Strayer & Malcom, 2018) are well established, but to the best of our knowledge, the effects of *D. polymorpha* on *P. complanata* have not yet been analyzed.

The core objective of this study was to characterize the depth‐ and size‐distribution of endangered *P. complanata* from Lake Siecino in relation to the loading with invasive *D. polymorpha*. Specifically, we hypothesized that (a) *P. complanata* would have density maxima at places with lotic conditions in the lake, differing from the distribution of other species such as *Unio tumidus*, (b) specimens of *P. complanata* would be less heavily fouled by *D. polymorpha* than other native mussel species due to their burrowing behavior, but (c) fouling of *P. complanata* with *D. polymorpha* would result in adverse effects on *P. complanata* as evident from shell deformities and difficulties of specimens to anchor and burrow into the substrate.

## 2 MATERIAL AND METHODS

### 2.1 Study area

**FIGURE 1:**
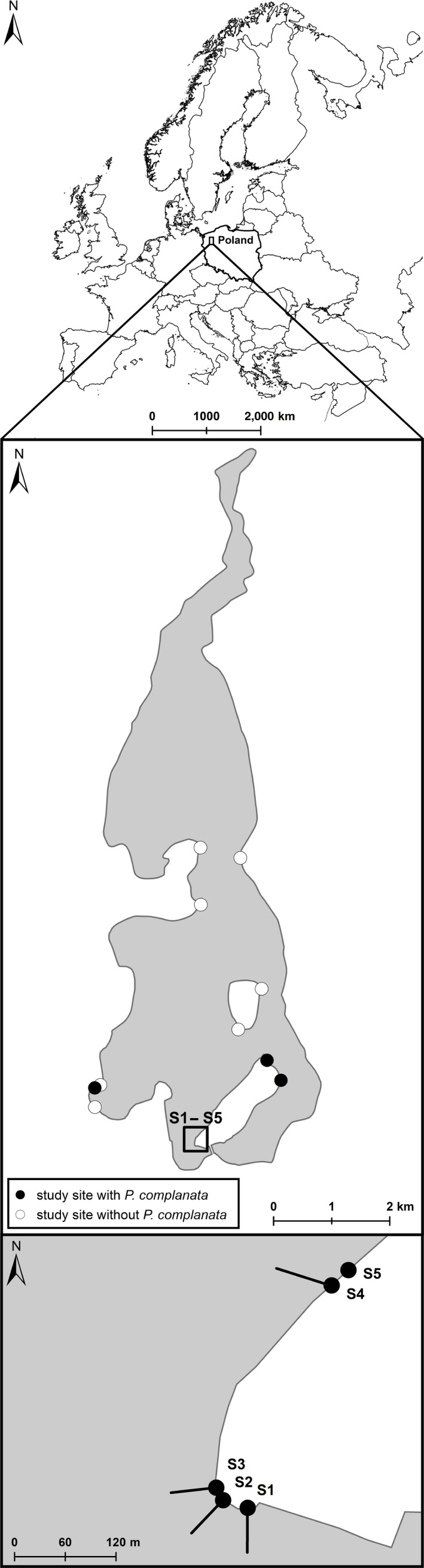
Location and map of Lake Siecino with the study sites; circles filled in black color indicate sites with *Pseudanodonta complanata*, white circles those without

The study was carried out in Lake Siecino in northern Poland (Figure 1). The lake is of glacial origin, narrow, and elongated in the north–south direction, with a maximum length of 7.4 km and a maximum width of 2.4 km; the surface area of the lake is 743 ha, the average depth is 14.2 m, and the maximum depth is 44.2 m. The lake is surrounded by forests in the south and by open areas in the north. Bottom sediments are fine‐grained, with sand and gravel predominating in its southern part, and muddy substrates in the north, reflecting the geological profile of the area (Lewandowski, Heliasz, & Chybiorz, 2006). Human impact on the lake is rather limited, and water quality is classified as “good” according to the Polish assessment system for surface water bodies (Wojewódzki Inspektorat Ochrony Środowiska w Szczecinie, 2017).

### 2.2 Physicochemical properties of the water

We assessed the physicochemical parameters of the water with a WTW Multi 3430 equipped with a IDS depth probe with oxygen, pH and conductivity (Xylem Analytics Germany Sales GmbH & Co. KG) lowered to the depth of 13 m. The measurements were taken once in the area of the highest depth of the lake (N 53.615994, E 16.024083) on 06 June 2018 at 15:40, and once in the area approximately 100 m offshore from the site where most *P. complanata* were found (N 53.587288, E 16.019255) on 07 June 2018 at 10:00.

The values of physicochemical water parameters were similar at both measurement sites. Water temperature decreased from above 20°C in the epilimnion to below 10°C in the hypolimnion, with a distinct thermocline between 5 and 7 m. Oxygen supersaturation of 106%–109% (9.4 mg/L) was recorded in the epilimnion, above 120% (12.6 mg/L) at the metalimnetic maximum, and 80.6% (9.8 mg/L) at a depth of 13 m. The water was slightly alkaline (pH range: 7.7–8.8), with an electrical conductivity between 238 and 244 μS/cm related to 25°C.

### 2.3 Mussel collection and documentation

Mussel survey was carried out on 4–8 June 2018 by qualified scientific divers (PH, HI, JM, FM, MvW, SZ) following the German Rules for Safety and Health Protection for the Operation of Scientific Divers (GUV‐R 2112). We found an abundant population of *P. complanata* at the southwestern side of the southern island and chose this place as the main study area. Four transects (study sites S1–S4), were laid out with a measuring tape by swimming in the direction of the respective course down to a depth of 10 m. Two transects were in the places of the highest densities of *P. complanata* (S2: N 53.587925, E 16.020294, course 245° and S3: N 53 587971, E 16.020204, course 260°) and two in the edge zones of this abundant occurrence (S1: N 53.587876, E 16.020526 course 180°, and S4: N 53.589267; E 16.021352, course 280°). Data collected in these transects constitute the core material of this study. Transects were documented by taking underwater pictures every 2 m (Nikon COOLPIX W300, Nikon GmbH). Depth profiles were established by taking bottom length measurements at every 1‐m water depth of the transects.

All mussels found on one searching occasion in a shallow, flat, and regularly raked bathing area (S5: N 53.589408; E 16.021473) were additionally included in the analyses not involving mussel depth distribution. To provide background information and to explore habitat conditions at places of *P. complanata* occurrence, we conducted reconnaissance surveys at ten preselected sites in other parts of the lake, with 10–20 min searches in each. All sites were photographed and examined for sediment type and unionid abundance. Water currents perceivable while diving were noted. Mussels found during the reconnaissance surveys were assigned to species and counted, but were not included in the analyses. Handheld Garmin GPS receivers were used to document the position of the study sites.

At sites S1–S4, all live unionids found to a sediment depth of approximately 5 cm in 2 m wide transects were collected by two concurrently working divers. Underwater, each mussel was placed in a separate individually marked plastic zipper bag to facilitate the documentation of the collection depth and to keep all attached zebra mussels. On the surface, the mussels were placed in cool lake water and were documented on‐site. Each unionid was photographed with all zebra mussels attached and after their removal. Species identification was based on external shell characteristics and was validated by genetic analysis (Zieritz, Gum, Kuehn, & Geist, 2012). To calculate mussel densities, we projected the collection depth of each animal onto the depth profile of the transect in which it was found, so that the densities expressed as the number of individuals per 1 m^2^ represent the actual mussel counts in every 0.5 m section of the 2 m wide transects. In order to calculate the load of *D. polymorpha* per individual unionid mussel and dreissenid: unionid mass ratios (Ricciardi, 2003), the blotted total wet mass (tissue and shell) of each unionid and of zebra mussels attached to it was obtained to the nearest 0.1 g. Unionid length, height, and width were measured using digital calipers to the nearest 0.1 mm, and age was estimated by counting external growth rings. Afterward, the unionid mussels were returned to places of collection.

### 2.4 Bottom sediments

We collected sediment cores with 30 cm long transparent plastic tubes from 2, 6, and 10 m depths at study sites S1–S4 and from 0.5 m depth at the study site S5. The sediment layers were classified into sand, mud, and soft organic top layer, according to their texture, color, and appearance, and their thickness was measured with the open‐source image editor GIMP 2.10.6. To the depth of approximately 6 m, the top sediments consisted mostly of sand or sand with muddy and organic inclusions and a thin layer of soft organic material. At greater depths, the compact sand layer was overlaid with a muddy organic layer. At the study site S5 the sediment consisted of only sand.

### 2.5 Statistical analysis

The differences in mussel density, shell length, and the load of *D. polymorpha* were tested with the Mann–Whitney test due to non‐normally distributed data. The interrelation between the load of *D. polymorpha*, shell length, age, and density of *P. complanata* were analyzed by principal component analysis (PCA). Additionally, correlations between densities of *P. complanata* and *D. polymorpha* loading as well as between size of *P. complanata* and *D. polymorpha* loading were analyzed. All analyses were carried out using XLStat 2017 (Addinsoft).

## 3 RESULTS

### 3.1 Mussel occurrence and depth distribution

Altogether six species of freshwater mussels were found in the lake: non‐native *D. polymorpha* and five species of native unionids: *Anodonta cygnea*, *Anodonta anatina*, *P. complanata*, *Unio pictorum*, and *U. tumidus*. *Pseudanodonta complanata* was most dominant, contributing to 91% of unionids collected at sites S1–S5 and to 56% of unionids found in the other parts of the lake (Table 1).

**TABLE 1:** Number of mussel individuals collected in Siecino Lake at the main study sites (S1–S5) and the reconnaissance survey sites (RS)

	S1	S2	S3	S4	S5	RS
*Anodonta anatina*	0	2	0	0	0	0
*Anodonta cygnea*	0	1	0	0	0	1
*Pseudanodonta complanata*	12	187	56	10	6	15
*Unio tumidus*	0	8	11	2	4	10
*Unio pictorum*	0	0	0	0	0	1

Note

Sites S1–S4 represent full‐depth profiles, S5 represents a shallow site with 0.6–0.7 m water depth.

**FIGURE 2:**
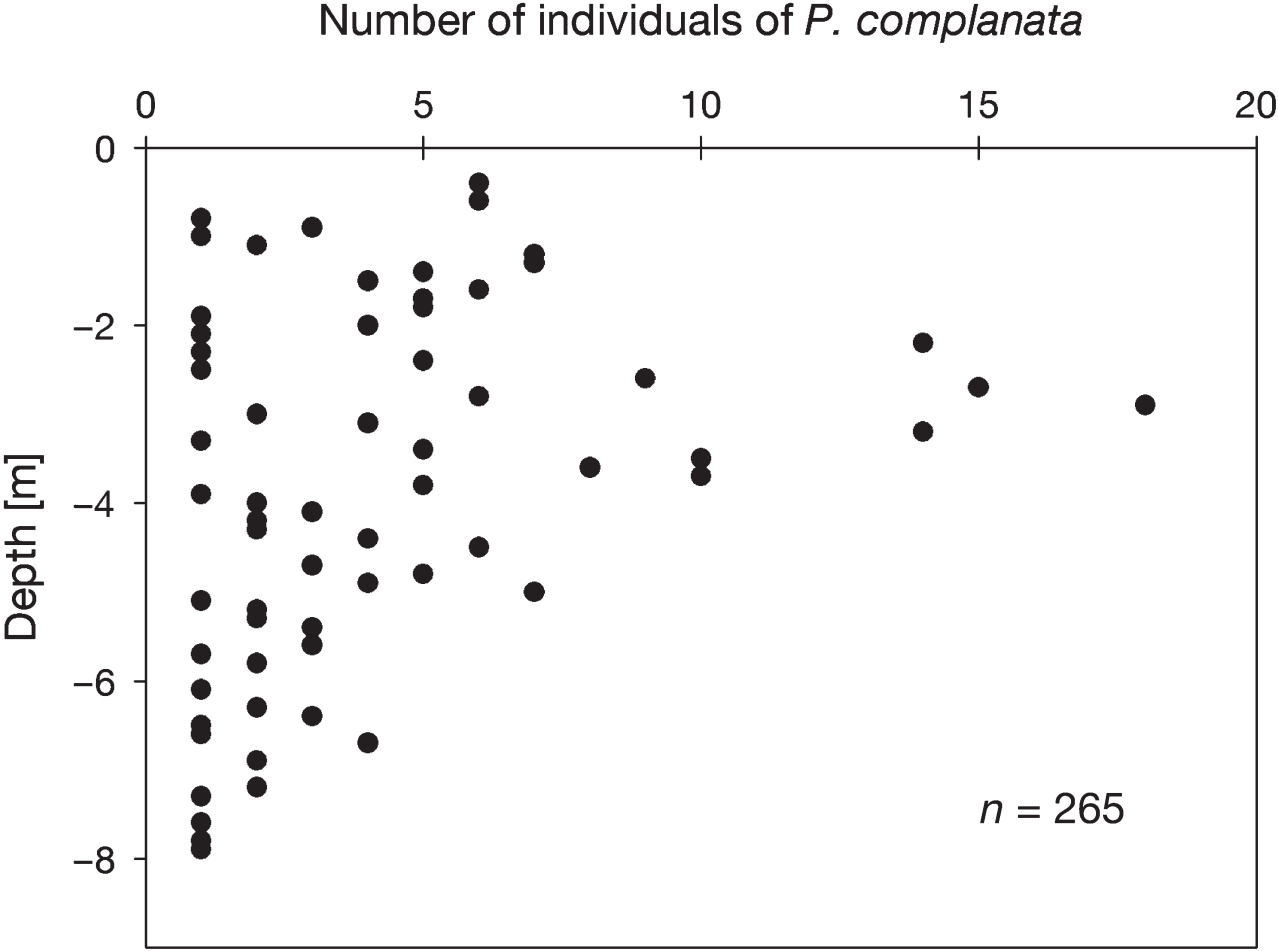
Depth distribution of *Pseudanodonta complanata* in the main study area. Number of individuals of *P. complanata* represents the number of individuals collected at specified depths over the study sites S1–S4

In the main study area (S1–S4), living *P. complanata* were found at water depths between 0.4 and 7.9 m (Figure 2). The highest occurrence was observed at around 3 m depth, with 51.3% of all individuals found between 2.0 and 4.0 m, and 75.9% between 1.5 and 5.5 m depth. The average density in the occupied depth range (0–8 m) was 1.3 ind/m^2^. An area of highest local densities (13–26 ind/m^2^) was observed at a water depth of 2.6–3.8 m at the site S2. Densities and individual shell lengths were higher on sandy than muddy substrates (Mann–Whitney test, *p* < .001, and *p* < .01, respectively). Distinct underwater currents were observed at the sites of the most abundant occurrence of *P. complanata*, that is, study sites S2 and S3. During the reconnaissance survey, *P. complanata* was found at three out of 10 sites. In all three, the bottom was sandy, and in two, noticeable underwater currents were observed. The bottom at sites without *P. complanata* was densely covered by rooted macrophytes or was soft and consisted of a thick layer of organic debris mostly containing reed and leaf remains.

Other unionids showed different patterns of depth distribution, indicating a preference for more shallow water. In *U. tumidus*, 67% of individuals collected at sites S1–S4 (*n* = 21) occurred at a depth of less than 1 m, 29% between 1 and 4 m and the maximum depth at which *U. tumidus* was found was 5.3 m. The three *Anodonta* individuals were found between 1 and 3.5 m depth.

**FIGURE 3:**
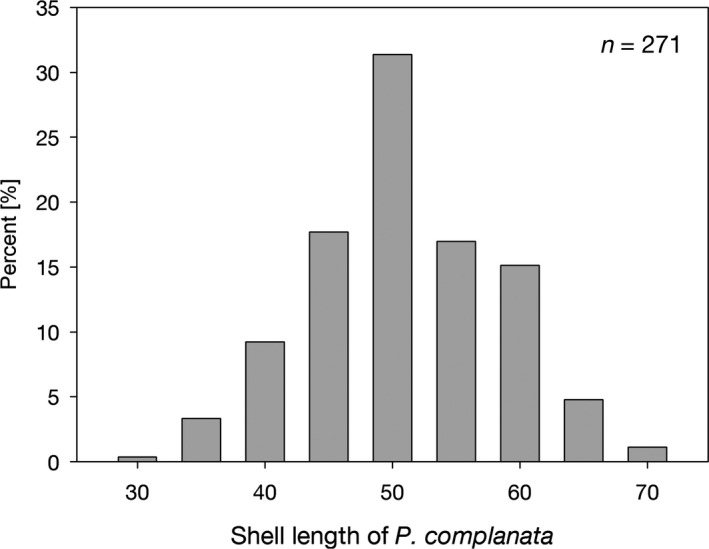
Shell length distribution in *Pseudanodonta complanata* at sites S1–S5

In *P. complanata*, the mean shell length (± *SD*) was 53.3 ± 7.5 mm, ranging from 33.6 to 72.8 mm, and the mean mussel age was 6.1 ± 1.7 years, ranging from 3 to 12 years. Individuals of medium size and age predominated, with low frequencies of small (young) and large (old) individuals (Figure 3). A similar pattern of highest frequencies of medium‐sized individuals was observed in *U. tumidus*. Measurements of all mussels are available via dryad (https://doi.org/10.5061/dryad.12jm63xtr).

Throughout the main study area (sites S1–S4) more dead shells than live unionid mussels were observed, including *A. anatina*, *A. cygnea*, *P. complanata,* and *U. tumidus.* Among the dead shells of *A. anatina* and *A. cygnea*, there were shells belonging to large size classes (shell lengths > 100 mm), not encountered among living individuals in this study (maximum shell length, 65 mm).

### 3.2 Effects of *D. polymorpha*

**FIGURE 4:**
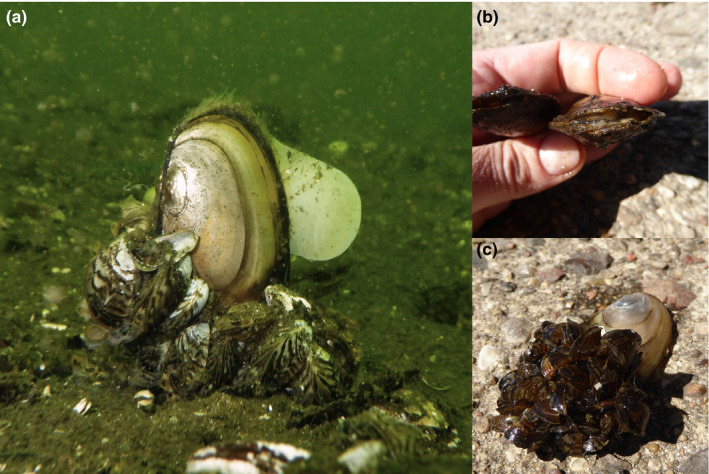
Photographic documentation of the infestation of *Pseudanodonta complanata* by *Dreissena polymorpha*. (a) Living *P. complanata* toppled over by the weight of attached *D. polymorpha*; (b) shell deformation; (c) heavily infested individual

*Dreissena polymorpha* occurred throughout the lake, fouling the available hard substrates, which included live and dead unionid shells. Based on our observations, attachment of *D. polymorpha* to *P. complanata* started at the posterior end, and ongoing attachment of additional individuals resulted in the formation of druses of various sizes (Figure 4). Thick layers of dead *D. polymorpha* shells were found below 5–7 m water depth.

**FIGURE 5:**
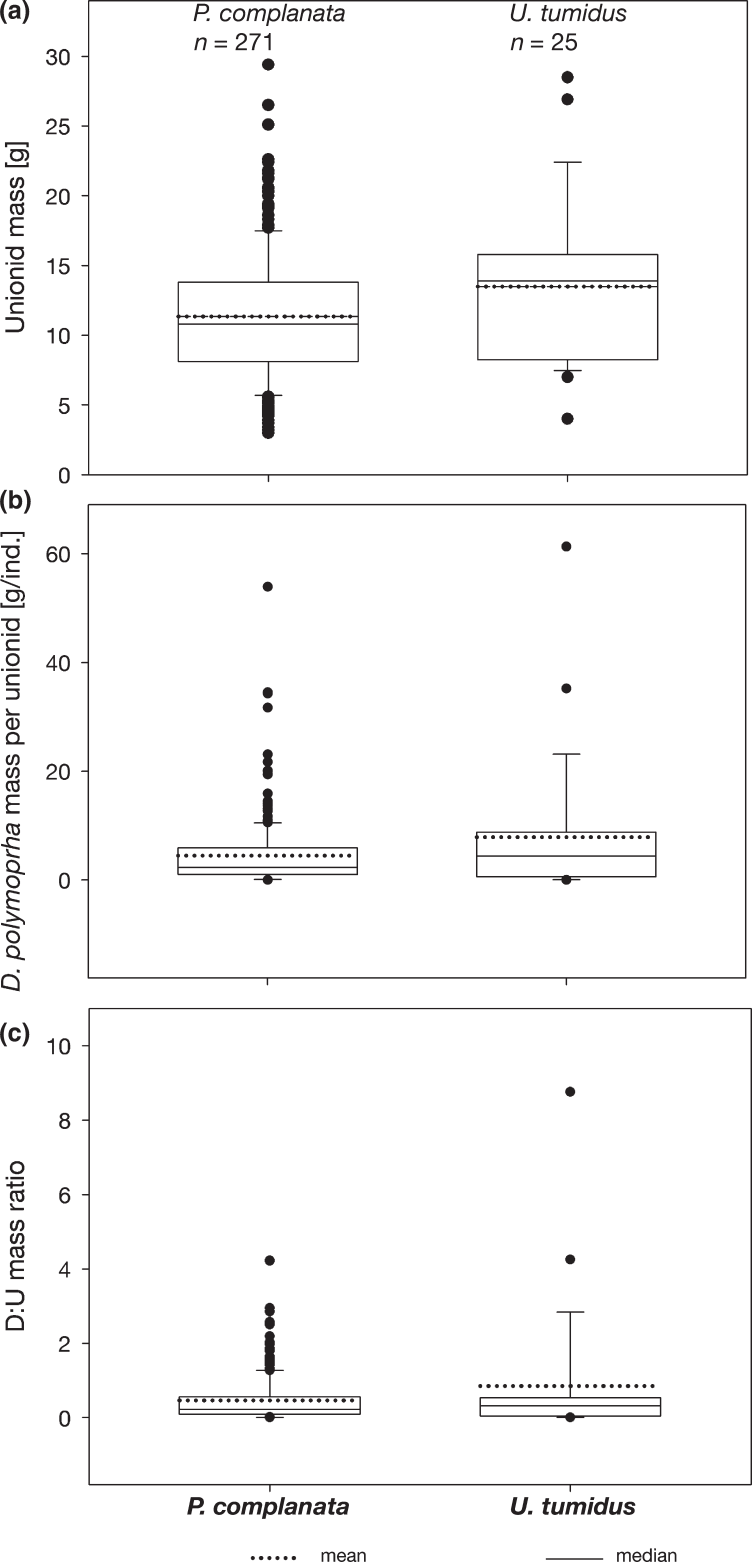
Unionid total wet mass (a), total wet mass of *Dreissena polymorpha* per unionid (b), and dreissenid: unionid (D:U) mass ratios (c) in *Pseudanodonta complanata* and *Unio tumidus* over the study sites S1–S5

**FIGURE 6:**
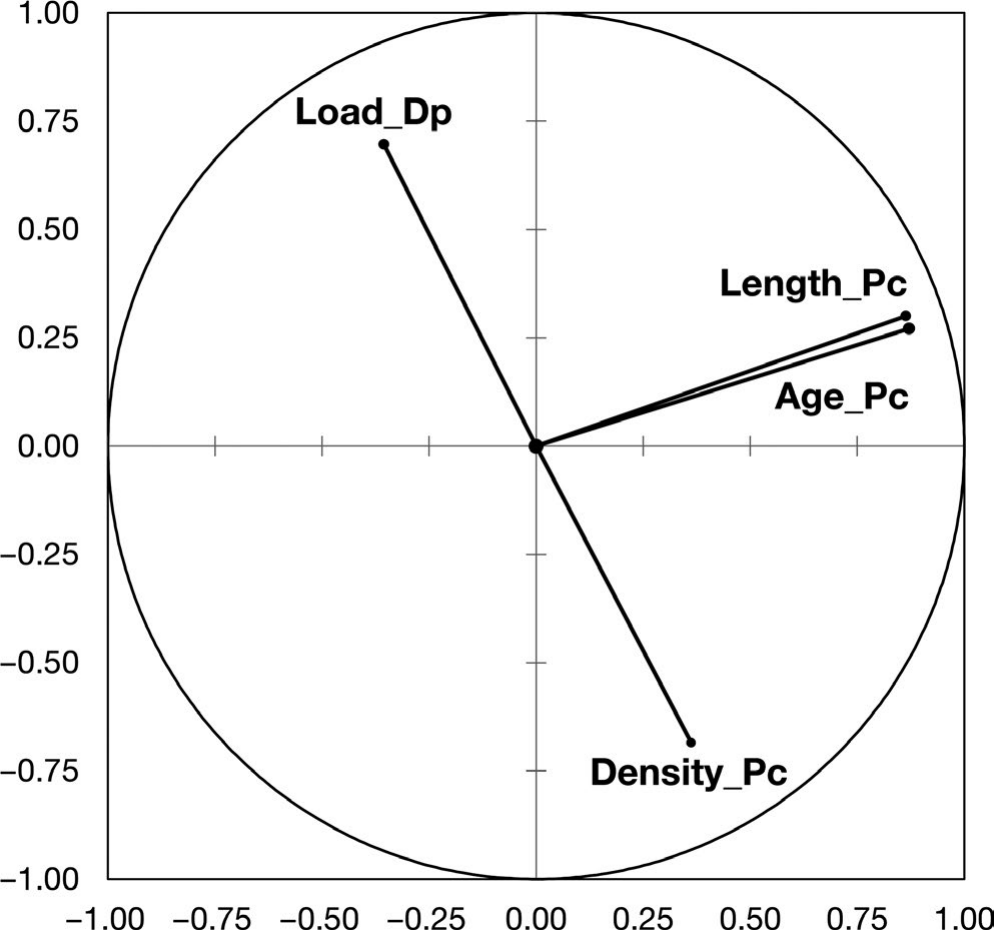
PCA of the interrelations between the load of *Dreissena polymorpha* (Load_Dp), shell length (Length_Pc), age (Age_Pc), and density of *Pseudanodonta complanata* (Density_Pc)

**FIGURE 7:**
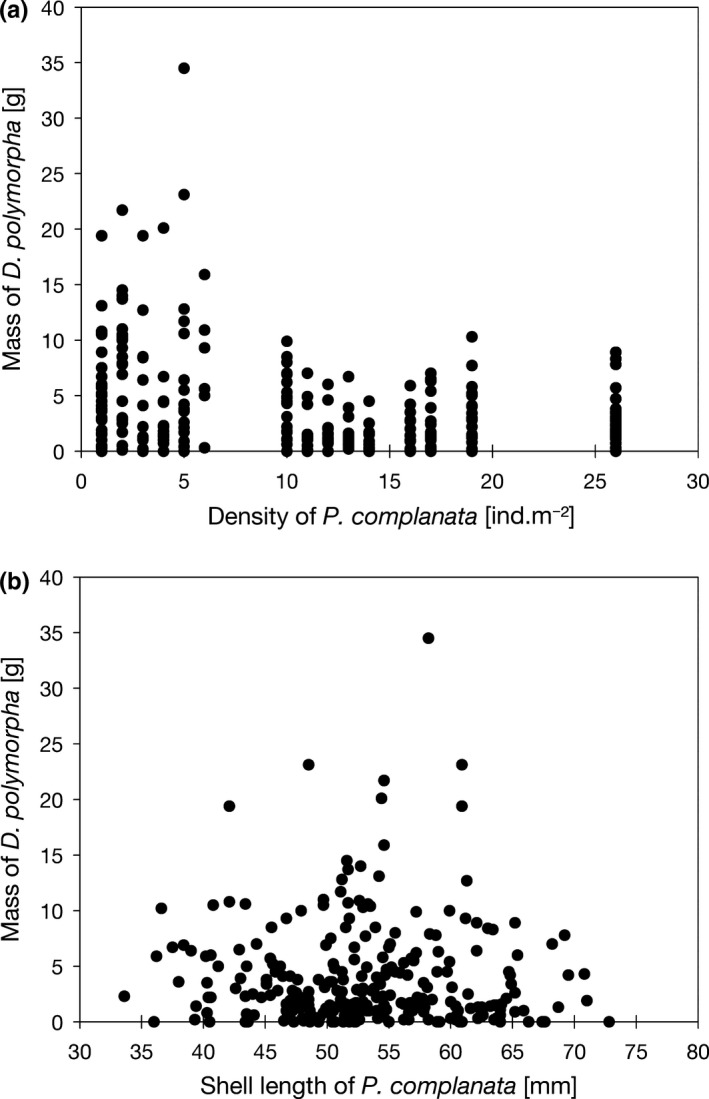
(a) Loading of native *Pseudanodonta complanata* with invasive *Dreissena polymorpha* [g per individual] in relation to the density of *P. complanata*; (b) loading of native *P. complanata* with invasive *D. polymorpha* [g per individual] in relation to the length of *P. complanata*

At study sites S1–S5, 91% of *P. complanata*, 92% of *U. tumidus* and all three individuals of *Anodonta* were fouled by *D. polymorpha*. The dreissenid: unionid mass ratio (mean ± *SD*; maximum) was 0.43 ± 0.56; 4.22 and 0.86 ± 1.87; 8.76 in *P. complanata* and *U. tumidus*, respectively. The difference between species was statistically significant (Mann–Whitney test, data arcsin transformed, *p* < .01) (Figure 5). The PCA of the interrelation between the load of *D. polymorpha*, shell length, age, and density of *P. complanata* showed a total loading on the first two axes of 72% (Figure 6, Table 2). The load of *D. polymorpha* increased with the decreasing density of *P. complanata* but was not correlated with its length or age (Figures 6 and 7). Highest fouling intensity up to 34.5 g of *D. polymorpha* per *P. complanata* was observed at or below a threshold density of six *P. complanata* m^−2^, whereas no loadings > 10.3 g *D. polymorpha* per host mussel occurred at densities of *P. complanata* ≥ 10 ind/m^2^ (Figure 7a). In contrast to our expectation, larger mussels with presumably longer exposure time to *D. polymorpha* colonization did not have greater mass of *D. polymorpha* on their shells compared to smaller ones (Figure 7b).

**TABLE 2:** Eigenvalues and loading of the calculated principal components

	F1	F2	F3	F4
Eigenvalue	1.762	1.118	0.797	0.323
Variability (%)	44.043	27.946	19.936	8.076
Cumulative %	44.043	71.988	91.924	100.000

Two impacts of *D. polymorpha* were directly observable. First, many individuals were not able to anchor deeply because of the encrustation with *D. polymorpha*, and some still living, fouled *P. complanata* were lying on the lake bottom, toppled over by the weight of attached *D. polymorpha*. These specimens were evidently not capable of returning to their normal position. Second, shell deformities in *P. complanata* were common and often strong (Figure 4).

## 4 DISCUSSION

This study characterizes an exceptionally dense population of *P. complanata*, highlighting the specific conditions under which this species can also occur in lakes. Second, it documents the impacts of *D. polymorpha* on this endangered species, not only through competition, but also through shell deformation and loss of anchoring in the substrate.

### 4.1 The occurrence of *P. complanata* in Lake Siecino

*Pseudanodonta complanata* typically occurs in lotic environments (Killeen et al., 2004; Piechocki & Wawrzyniak‐Wydrowska, 2016). In Lake Siecino, a combination of regional and local air circulation (Kirschenstein, 2004), specific lake morphology, and the pattern of distribution of forested and open land in the adjacent area contribute to strong winds blowing regularly along the long axis of the lake, causing a substantial movement of surface waters and strong returning currents. Additional acceleration of water flow occurs along the shores of two islands, where the lake narrows, and the bottom falls off steeply. Thus, in some parts of Lake Siecino conditions are similar to a lotic system, and indeed, *P. complanata* occurred most abundantly around the southernmost island, at sites with the strongest predicted and observed underwater currents. Other patterns in the occurrence of *P. complanata* in Lake Siecino indicate its preference for sandy bottom without organic debris and for places with steep bottom slopes. Although *P. complanata* can occur to a depth of 11 m (Van Damme, 2011), 76% of individuals in our study occurred above 5.5 m, that is, above the current thermocline. This is possibly related to higher productivity, and thus higher food availability in the epilimnion. Additionally, steep banks facilitate mussel migration along thermal gradients, which might be especially important in thermally stratified water bodies.

### 4.2 Impacts of *D. polymorpha* fouling on unionid mussels

In Lake Siecino, 91% of unionids were fouled by *D. polymorpha*. The mean ratio of the mass of attached *D. polymorpha* to that of their unionid host was 0.43 in *P. complanata* and 0.86 in *U. tumidus*. This variable is a good predictor of unionid population mortality (Ricciardi, 2003), and the values observed in our study indicate that fast population declines are to be expected. Positive correlation between fouling intensity and field densities of *D. polymorpha* has been documented before (Burlakova, Karatayev, & Padilla, 2000; Lewandowski, 1976; Ricciardi, Whoriskey, & Rasmussen, 1995). Our study shows that fouling intensity was negatively correlated with unionid density: the mass of *D. polymorpha* per individual *P. complanata* was highest at the lowest density of this unionid, with the pattern following a threshold‐distribution rather than a linear relationship. This is explainable in view of the preferential settlement of *D. polymorpha* on unionids (Lewandowski, 1976; Ricciardi, Whoriskey, & Rasmussen, 1996) and of the benefits it gains from this substrate choice (Hörmann & Maier, 2006; Pilotto, Sousa, & Aldridge, 2016). As the harmful effects of *D. polymorpha* on a unionid host are proportional to the degree of fouling (Haag, Berg, Garton, & Farris, 1993; Sousa et al., 2011), the impacts of the invader are likely to increase with decreasing unionid densities, leading to accelerated population declines.

Deeply burrowing species, such as *P. complanata*, are usually less vulnerable to the settlement of *D. polymorpha* larvae than species with larger parts of their shells exposed (Arter, 1989; Marszewska & Cichy, 2018; Urbańska, Andrzejewski, Giersztal, & Golski, 2018), but remain vulnerable to adult *D. polymorpha* actively searching for suitable substrates (Hallac & Marsden, 2001; Lewandowski, 1976). Once the first *D. polymorpha* attach, their individual growth gradually pulls the unionid out of the sediment and exposes its shell to further colonization, which in *P. complanata* is facilitated by its strongly flattened and smooth shell. Additionally, *P. complanata* occurs preferentially on sand or gravel, where fouling by *D. polymorpha* is usually higher (Burlakova et al., 2000; Dzierżyńska‐Białończyk, Jermacz, Maćkiewicz, Gajewska, & Kobak, 2018) and more detrimental than on muddy bottom (Bowers & De Szalay, 2004; Nichols & Wilcox, 1997). In most individuals in this study, the distance between the surface of the sediment and the highest part of the shell was equal to the part of the shell encrusted with *D. polymorpha*; *P. complanata* was not able to anchor deeply in the sediments, but instead was pulled out of the sand by the growth of attached *D. polymorpha*. The result was toppling over of the mussel, consequently leading to its death.

As in *P. complanata* normally only siphons extend above the sediment surface (Saarinen & Taskinen, 2003), any infestation by *D. polymorpha* begins in their direct vicinity, and will interfere with its shell growth, especially that the shell in this species is thin and delicate. Indeed, we observed strong deformations of the posterior end of the shell in almost all *P. complanata* individuals. Such shell deformities as an effect of *D. polymorpha* fouling have also been observed in other unionid mussels (e.g., Mackie, 1991). They can reduce the mussels' ability to close their shells during adverse conditions, impair their filtering activity and render them more susceptible to predation and parasites.

The frequencies of small (young) individuals were low, indicating low recent recruitment, although an underrepresentation of smaller individuals can be due to their lower detectability. However, the frequencies of individuals in the large size classes were also low and an underrepresentation of these due to a sampling bias is highly unlikely. As large mussel individuals tend to be more susceptible to decreased food levels than small ones (Nalepa, Hartson, Gostenik, Fanslow, & Lang, 1996), the invasion of *D. polymorpha* can affect disproportionally unionids in the larger size classes. This is also indicated by the absence of large living *A. anatina* and *A. cygnea*, while dead shells of large specimens of these species were present on the lake bottom. For *P. complanata,* it takes about 4 years to reach maturity, and it is a long‐term brooder; *that is*, it holds glochidia for most of the year and releases them in spring or summer. The number of glochidia increases exponentially with mussel length, and in natural populations, the largest size classes comprise the most fecund females and a high proportion of males (McIvor & Aldridge, 2007). Thus, the loss of the largest mussels can lead to strongly decreased reproductive output of the population and add to the secondary effects of *D. polymorpha* invasion.

Due to its deep‐burrowing behavior, *P. complanata* has been suggested to be the least susceptible to colonization among European unionids (Sousa et al., 2011). On the other hand, species with flattened, thin and smooth shells, living in firm compacted substrates, maturing late, and with long brooding time are especially vulnerable to *D. polymorpha* attack (Gillis & Mackie, 1994; Haag et al., 1993; Hallac & Marsden, 2000; Nalepa, 1994; Zanatta et al., 2015). *Pseudanodonta complanata* fulfills all these other criteria of vulnerability, and our study shows that when almost no other unionids are present, and hard substrata are generally scarce, it can be intensively exploited with strong negative effects on individual survival, fitness, and population sustainability.

Invasion of *D. polymorpha* can alter community structure of unionid mussels by causing differential mortality and reductions in fitness among unionid species (Gillis & Mackie, 1994; Haag et al., 1993; Nalepa et al., 1996; Strayer & Malcom, 2018; Zanatta et al., 2015). Our study documents the occurrence of all five unionid species for which Lake Siecino provides suitable habitats, albeit at extremely low abundances; dead shells found on the lake bottom document their relatively recent more abundant occurrence. Although we do not have a direct evidence of this, strong declines of other species, which are usually more susceptible to *D. polymorpha* fouling than *P. complanta* (Sousa et al., 2011), may have preceded its currently observed massive infestation; this is also suggested by the level of fouling significantly higher in *U. tumidus* than in *P. complanata*. Such sequential mortality would not have been unprecedented (Strayer & Malcom, 2018; Zanatta et al., 2015); in Europe similar sequences of strong population declines were observed in Lake Hallwil, Switzerland (Arter, 1989) and at Barden Lake, UK (Aldridge, Elliott, & Moggridge, 2004). Contrary to the reports in which the effect of *D. polymorpha* invasion on unionid mortality was difficult to disentangle from other factors such as eutrophication and pollution (Arter, 1989; Lewandowski, 1991), there are no indications of the deterioration of water quality in Lake Siecino, and thus, the invasion of *D. polymorpha* is the most probable cause of unionid mortality.

The time for the invasion of *D. polymorpha* to play out differs among ecosystems and can take many years (Strayer & Malcom, 2018). In our study, it has coexisted with native mussels for more than 40 years, but its effects, even if not fast, are clearly detrimental. Although European unionids appear to be less sensitive to *D. polymorpha* than American ones (Karatayev, Burlakova, & Padilla, 1997), their strong declines or local extinctions following the invasion of *D. polymorpha* have been recorded (Aldridge et al., 2004; Arter, 1989; Lewandowski, 1991; Lucy, Burlakova, Karatayev, Mastitsky, & Zanatta, 2014), and the negative impact of *D. polymorpha* fouling on European unionids is well documented (Bódis, Tóth, & Sousa, 2014; Sousa et al., 2011). In spite of this, an opinion prevails that *D. polymorpha* invasion in European waters does not have long‐lasting negative effects. It is even considered as a suitable tool for the enhancement of water quality, and its spread or encouragement of increased abundance is being justified and promoted, both in North America (e.g., Strayer, 2009) and Europe (our own observations). Clear policies preventing this practice need to be developed. Also further accidental spread of this species should be limited, and to this end, well‐designed and well‐targeted educational campaigns can be most effective. From a perspective of conservation management of endangered unionid mussels, active removal of *D. polymorpha* from infested individuals may be a feasible option (Hallac & Marsden, 2000, 2001), yet needs to be verified in terms of strength and duration of this effect.

## 5 CONCLUSIONS

In this study, we documented an exceptionally large population of *P. complanata* inhabiting a postglacial lake. Specific local conditions resulting in regularly occurring water currents provide lotic habitats and can explain its abundance; its occurrence in other lakes with similar characteristics can be expected. Infestation of unionid mussels with the invasive *D. polymorpha* was prevalent. The effect of *D. polymorpha* on *P. complanata* was adverse, as evidenced by common and strong shell deformities, and the inability of the infested individuals to burrow in the sediments. Although deep‐burrowing species of unionids are relatively less vulnerable to the settlement of *D. polymorpha*, other characteristics of *P. complanata* (strongly flattened and smooth shell, preference for sandy bottom, late maturation and long brooding time) render it susceptible to the invasion of *D. polymorpha* which should therefore be regarded as an important threat to the survival of this rare and endangered species. Our study contributes to the growing evidence documenting detrimental effects of *D. polymorpha* on native unionids in Europe.

## CONFLICT OF INTEREST

The authors declare that there is no conflict of interest.

## AUTHOR CONTRIBUTION

**Małgorzata Ożgo:** Conceptualization (lead); data curation (equal); formal analysis (lead); funding acquisition (lead); investigation (lead); methodology (equal); project administration (equal); resources (equal); supervision (equal); validation (lead); visualization (lead); writing – original draft (lead); writing – review and editing (lead). **Maria Urbanska:** Conceptualization (lead); data curation (equal); investigation (supporting); methodology (equal); supervision (equal); writing – review and editing (supporting). **Philipp Hoos:** Data curation (equal); investigation (supporting); methodology (supporting); writing – review and editing (supporting). **Hannes K. Imhof:** Data curation (equal); investigation (supporting); methodology (equal); visualization (supporting); writing – review and editing (supporting). **Małgorzata Kirschenstein:** Investigation (supporting); writing – review and editing (supporting). **Julia Mayr:** Data curation (equal); investigation (supporting); methodology (supporting); writing – review and editing (supporting). **Florian Michl:** Data curation (equal); formal analysis (supporting); investigation (supporting); methodology (equal); writing – review and editing (supporting). **Rafal Tobiasz:** Data curation (equal); investigation (supporting); methodology (supporting); resources (supporting); writing – review and editing (supporting). **Marie von Wesendonk:** Data curation (equal); investigation (supporting); methodology (supporting); writing – review and editing (supporting). **Stefan Zimmermann:** Data curation (equal); investigation (supporting); methodology (equal); writing – review and editing (supporting). **Juergen Geist:** Conceptualization (lead); data curation (equal); formal analysis (lead); funding acquisition (lead); investigation (lead); methodology (equal); project administration (equal); resources (equal); supervision (equal); validation (lead); Visualization (supporting); writing – original draft (lead); writing – review and editing (lead).

## Data Availability

All raw data are accessible via dryad (https://doi.org/10.5061/dryad.12jm63xtr).
